# Prediction of Extracellular Proteases of the Human Pathogen *Helicobacter pylori* Reveals Proteolytic Activity of the Hp1018/19 Protein HtrA

**DOI:** 10.1371/journal.pone.0003510

**Published:** 2008-10-23

**Authors:** Martin Löwer, Christiane Weydig, Dirk Metzler, Andreas Reuter, Anna Starzinski-Powitz, Silja Wessler, Gisbert Schneider

**Affiliations:** 1 Goethe-University, Institute of Cell Biology and Neuroscience / CMP, Frankfurt am Main, Germany; 2 Junior Research Group, Paul-Ehrlich Institute, Langen, Germany; 3 Goethe-University, Institute of Computer Science, Frankfurt am Main, Germany; 4 Paul-Ehrlich Institute, Department of Allergology, Langen, Germany; Duke University Medical Center, United States of America

## Abstract

Exported proteases of *Helicobacter pylori* (*H. pylori*) are potentially involved in pathogen-associated disorders leading to gastric inflammation and neoplasia. By comprehensive sequence screening of the *H. pylori* proteome for predicted secreted proteases, we retrieved several candidate genes. We detected caseinolytic activities of several such proteases, which are released independently from the *H. pylori* type IV secretion system encoded by the *cag* pathogenicity island (*cag*PAI). Among these, we found the predicted serine protease HtrA (Hp1019), which was previously identified in the bacterial secretome of *H. pylori*. Importantly, we further found that the *H. pylori* genes *hp1018* and *hp1019* represent a single gene likely coding for an exported protein. Here, we directly verified proteolytic activity of HtrA *in vitro* and identified the HtrA protease in zymograms by mass spectrometry. Overexpressed and purified HtrA exhibited pronounced proteolytic activity, which is inactivated after mutation of Ser205 to alanine in the predicted active center of HtrA. These data demonstrate that *H. pylori* secretes HtrA as an active protease, which might represent a novel candidate target for therapeutic intervention strategies.

## Introduction

The mucosal epithelium in the human stomach forms the first barrier that prevents infiltration of pathogens into the host organism. The human pathogen *H. pylori* developed efficient strategies to colonize the gastric epithelium as a unique niche, where it induces the disruption of the epithelial layer contributing to inflammatory diseases (*e.g.* chronic gastritis, ulceration), mucosa-associated lymphoid tissue (MALT) lymphoma and gastric cancer in humans [1], [2]. More virulent *H. pylori* strains express a combination of key disease-associated virulence factors allowing successful colonization in the stomach [3]. Among those, *H. pylori* harbors *cag* pathogenicity island (*cag*PAI), which encodes a type IV secretion system (T4SS) to inject the bacterial CagA (cytotoxin-associated gene A) oncoprotein into host cells [4]. *In vitro*, translocated CagA can strongly enhance the disruption of intercellular adhesions [4], [5]. This process is believed to contribute to inflammation, carcinogenesis and invasive growth. Although the cellular aspects of CagA have been investigated intensively, the complex mechanisms of the actual interaction of *H. pylori* and the human epithelium are not fully understood yet.

Many pathogens developed elegant mechanisms for tissue destruction by secreting proteins with proteolytic activity. Exported bacterial enzymes can directly activate host *pro*-matrix-metalloproteinases (*pro*-MMPs) representing a biochemical efficient way for matrix degradation. An example is set by the wide range of proteases of the thermolysin family secreted by *Pseudomonas aeruginosa* and *Vibrio cholera* that activate *pro*-MMP-1, -8, and -9 [6]. It has been further observed that serine proteases associated with lipopolysaccharides can induce MMP-9 activity in macrophages [7]. MMP-9 cleavage was also detected by a secreted zinc metalloproteinase (ZmpC) from *Streptococcus pneumoniae*, which indicates that ZmpC may play a role in pneumococcal virulence and pathogenicity in the lung [8].

Proteases might also play a role in *H. pylori* pathogenesis, and protease secretion has already been described for this organism [9]. *H. pylori* sheds an unknown protease that efficiently degrades PDGF (platelet derived growth factor) and TGF-β (transforming growth factor beta), which can be inhibited with sulglycotide [10]. Some features present in the primary sequence of *H. pylori* virulence factor vacuolating cytotoxin A (VacA) are reminiscent of serine proteases [11], although the predicted proteolytic activity of VacA has not been detected yet. In 1997, a *H. pylori* metalloproteinase with a native molecular size of approximately 200 kDa was discovered, which was secreted when *H. pylori* was grown in liquid culture [12]. The authors hypothesized that surface expression of this metalloprotease activity may be involved in proteolysis of a variety of host proteins *in vivo* and thereby contribute to gastric pathology [12]. Importantly, *H. pylori* secretes a collagenase, encoded by *hp0169*, which might represent an essential virulence factor for *H. pylori* stomach colonization [13]. The predicted serine protease and chaperone HtrA (Hp1019) was previously identified as an extracellular protein of *H. pylori* [14], but its proteolytic role and substrates are still unknown.

As 658 of the 1,576 identified genes of the *H. pylori* genome [15] are annotated as “hypothetical” or as bearing a hypothetical function [16], we aimed at the identification of *H. pylori* genes possibly coding for secreted proteases by combining genomic data analysis with zymography. Indeed, we found that *H. pylori* secretes unknown proteins exhibiting caseinolytic activity. By calculating similarities to known proteases and using localization prediction methods, we inferred function and localization of these hypothetical *H. pylori* proteins. We also identified a sequencing error in the *hp1018* gene, which after correction encodes for a signal peptide for the putative serine protease HtrA (Hp1019). Eventually, we verified proteolytic activity of HtrA in biochemical approaches. The present study demonstrates the usefulness of sequence-based genome mining for potential drug targets representing one possible route for the prevention of matrix degradation of the mucosal epithelium by *H. pylori* and other pathogens.

## Results and Discussion

### *H. pylori* secretes caseinolytic proteases

**Figure 1:**
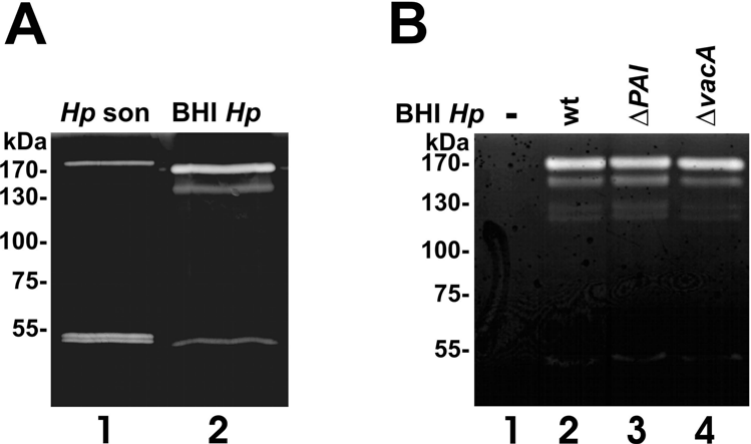
*H. pylori* secretes bacterial factors with caseinolytic activities. (A) The *H. pylori* strain 26695 was grown in protein-free BHI medium. After 48 hours, the bacteria were harvested and lysed by sonfication (*Hp* son). 30 µl aliquots of the supernatants (BHI *Hp*) and bacterial lysates were separated by casein zymography and analyzed proteolytic activities. (B) *H. pylori* strains wild type (P12, wt), ΔPAI, and ΔVacA were grown in protein-free liquid growth medium. 30 µl of the aliquots of the medium were analyzed in casein zymograms for proteolytic activities.

Data are accumulating that bacteria secrete proteases with functional roles in microbial pathogenesis, but knowledge of *H. pylori*-secreted proteases and their functions is still limited. To analyze whether *H. pylori* actually secretes proteases, we performed casein zymography to monitor proteolytic activity in the supernatants of *H. pylori* lysates (Figure 1A, lane1) and *H. pylori* culture medium (Figure 1A, lane 2). At least three casein-cleaving proteases were exported by *H. pylori* exhibiting apparent molecular weights of approximately 170 kDa, 140 kDa, and 50 kDa (Figure 1A, lane 2). Interestingly, the protein band pattern present in the supernatant of the *H. pylori* medium obviously differs from the equivalent *H. pylori* lysate (Figure 1A lanes 1). The detected 170 kDa protease present in the supernatant of *H. pylori* (BHI *Hp*) consistently migrated slightly faster than in the *H. pylori* lysate, while the 140 kDa protein was only present in the supernatant, but absent in the lysate of *H. pylori* (*Hp* son). In contrast to the double band detected in the lysates, we observed only a single proteolytic activity in the supernatant (Figure 1A, lanes 1–2). These data indicate that the export of the proteases might occur *via* active signal peptide-dependent translocation, rather than being an artifact of bacterial autolysis in the *H. pylori* liquid culture.

Since *H. pylori* encodes a well-described T4SS and the T4SS-independently secreted pathogenic factor VacA with a hypothesized protease function [11], we also included supernatants of isogenic *H. pylori* mutants which are deficient of T4SS and CagA (ΔPAI, Figure 1B, lane 3), or VacA (ΔVacA, Figure 1B, lane 4), and compared them with the *H. pylori* wildtype strain (wt, Figure 1B, lane 2) and *H. pylori*-free culture medium (-, Figure 1B, lane 1). Compared to the wildtype strain, the ΔPAI mutant showed the same secretion pattern of proteins with caseinolytic activity in the extracellular space suggesting that their secretion might occur independently from the T4SS (Figure 1B, lane 3). Although initial publications indicated a predicted serine protease activity of the pathogenic factor VacA [11], we can exclude a caseinolytic effect of VacA since the isogenic *vacA*-deficient *H. pylori* mutant showed a similar pattern of proteases (Figure 1B, lane 4). Gelatin zymographies were also performed by us and clearly demonstrated the lack of gelatinolytical *H. pylori* proteases (not shown). A positive result here would have demonstrated a closer link to matrix degaradation, as gelatin is a product of collagen, a major extacellular matrix protein.

So far, the identity of the detected *H. pylori* proteases was unknown. A previously described multi-metalloprotease-like complex secreted by *H. pylori* with a molecular weight of about 200 kDa [12] might be an explanation for the largest protein seen in the zymogram, since its size is four to six times greater than comparable proteases of other Gram-negative bacteria [12]. Also, protease DegP of *Escherichia coli*, which is a homolog of Hp1019 from *H. pylori*, was shown to form hexamers when crystallized [17]. Therefore, as zymography was performed under non-reducing conditions, the upper band(s) might result from smaller proteins forming a macromolecular complex.

### *In silico* genome screening for candidates of *H. pylori* secreted hypothetical proteases

Based on the finding that *H. pylori* actively secretes proteases, we then aimed to identify suitable candidates by *in silico* analysis. Thus, we compared the *H. pylori* proteome to a set of known proteases from various organisms using sequence alignment techniques. A reference set of known proteases containing 3,566 amino acid sequences was compiled from the UniProtKB/SwissProt database (version 6.7) [18], which served as queries for exhaustive pairwise alignment to genomic and protein sequence data of *H. pylori* strain 26695 with 1,576 annotated genes from the NCBI RefSeq database (accession number NC_000915) [16]. For the 1,576 putative *H. pylori* proteins, 75,524 local alignments were returned by the BLAST algorithm [19]. Alignments yielding an *E*-value≤0.5 were selected and divided into four classes:

Class A: alignments showing complete conservation of the active-site region,
Class B: alignments showing partial conservation of the active-site region,
Class C: alignments with proteases lacking an active-site annotation, and
Class D: all other alignments.

The latter (class D) were not further examined. Information about the localization of the active sites was retrieved from the feature tables of the respective SwissProt entries [18].

Then, we predicted protein localization using prediction systems, which are publicly available on the World Wide Web: SignalP [20], SecretomeP [21], Phobius [22], CELLO [23], PA-SUB [24], and PSORTb [25]. All systems are capable or explicitly designed to analyze amino acid sequences from Gram-negative bacteria. Alignments were selected for further examination when the corresponding predictions for a protein sequence matched one or more of the following criteria:

i) predicted extracellular localization (CELLO, PSORTb, PA-SUB),
ii) predicted signal peptide (SignalP),
iii) predicted signal peptide, but no transmembrane helices (Phobius), and a SecretomeP *score*≥0.5.

By filtering the alignments with respect to the active residues marked in the sequence of reference protease and the localization prediction, we obtained 47 class A, 39 class B and 32 class C proteins (*vide supra*) and their corresponding genes. The best-scoring alignments of those proteins to proteases of the reference set were manually inspected. Among those, nine genes have not been described to code for *H. pylori* proteases yet, but can be aligned with a statistically significant score to proteases of the reference protease sequences (Table 1). Interestingly, the putative translation products of genes *hp0289*, *hp0609*, and *hp0922* form a group of paralogs to VacA cytotoxin [26], which can be seen in a multiple sequence alignment (not shown). Structural similarities of VacA to extracellular IgA proteases of *Haemophilus influenzae* have been described previously [11]. Pairwise sequence identity of these VacA paralogs to VacA ranges between 25% and 30% which also include the C-terminal autotransporter sequence [27]. As this sequence is sufficient to transclocate the N-terminal part of the protein across the outer membrane − which is often followed by an autoproteolytic event to release the translocated part into the extracellular space [27] − it seems likely that some of these proteins possess a proteolytical function.

**Table 1:** Results of the Blastp search for proteases genes, of the localization prediction and calculated molecular weights (MW).

locus tag	description	matrix	*E*-Value	bitscore	% id.	class	Calculated MW (kDa)	CELLO	PA-SUB	Phobius	PSORTb	SecP	SignalP
hp0289	toxin-like outer membrane protein	blo62	0.009	38.9	22.3	A	31.12	EX	EX	+	EX	+	+
hp0506	conserved hypothetical secreted protein	blo80	8×10^−14^	72.8	40.7	A	45.97	OM	EX	1	U		+
hp0608	hypothetical protein	pam250	7×10^−4^	36.9	23.6	A	17.94	OM	−		IM		
hp0609	hypothetical protein	pam250	0.006	37.9	17.7	A	13.51	EX	EX, OM		U	+	
hp0657	processing protease (ymxG)	blo45	4×10^−9^	56.7	20.7	A	48.80	OM	EX	+	U		+
hp0922	toxin-like outer membrane protein	blo80	0.080	35.7	30.5	A	27.46	EX	EX	+	EX	+	
hp0980	conserved hypothetical secreted protein	pam30	3×10 ^−12^	65.5	38.6	A	11.41	PP	EX, IM	+	IM		+
hp1012	protease (pqqE)	blo62	1×10^−20^	95.5	23.8	A	50.33	OM, PP	EX	+	OM		+
hp1019	serine protease (htrA)	blo80	4×10^−82^	299	42.2	A	47.98	EX	EX, PP		PP	+	
hp1037	hypothetical protein	pam70	0.061	33.3	30.7	A	40.80	CP	CP, EX		CP		
hp1350	protease	blo80	7×10^−81^	295	39.7	A	50.55	CP	EX	+	U	+	+
hp1543	toxR-activated gene (tagE)	blo80	1×10^−16^	81.5	43.5	A	35.63	OM	EX	1	U		
hp1544	toxR-activated gene (tagE)	blo62	5×10^−8^	52.8	34.0	A	34.93	CP	EX,OM,PP	1	U		
hp1584	sialoglycoprotease (gcp)	blo80	1×10^−39^	158	36.4	C	37.80	CP	EX		EX		

Locus tags and descriptions were taken from the corresponding GenBank entries. The columns “matrix”, “*E*-Value”, “bitscore” and “% id.” list the alignment data of the according highest scoring alignment. The column “class” refers to our definition of alignment classes. Molecular weight was calculated by a program hosted on the ExPASy server. The columns “CELLO”, “PA-SUB”, and “PSORTb” give the classifications according to the prediction software. The column “Phobius” gives the number of transmembrane helices and a plus (+) sign if a signal peptide was found. Columns “SecP” and “SignalP” contain a plus sign for a SecretomeP output≥0.5 or prediction of a signal peptide, respectively. CP = cytoplasm, IM = inner membrane, PP = periplasm, OM = outer membrane, EX = extracellular, U = unknown.

Finally, although we could not detect caseinolytic activity of VacA in our casein zymography study, we cannot exclude an effect of VacA and its paralogs on other substrates *per se*. However, the alignments do not reveal conserved active site residues in VacA paralogs. Still they might represent autoproteolytic autotransporter proteins without common protease motifs which have been reported already [27]. Notably, their precursor proteins have a molecular weight of 136 to 311 kDa (according to SwissProt entries O25063, O25330 and O25579) which is in accordance with the molecular weights we observed in the zymography after a possible cleavage of the N-terminal signal peptide and the autotransporter sequence.

*H. pylori* harbors five genes that are described in the literature and/or database annotations to code for potential extracellular proteases (Table 1). Processing protease YmxG (Hp0657) and protease pqqE (Hp1012) are predicted to possess a signal peptide (Table 1) and to be extracellular or outer membrane-bound. The protease coded by the gene *hp1350* could be extracellular, as SecretomeP and PA-Sub vote for this localization and the existence of a signal peptide is also predicted (Table 1). The product of *hp1019*, which is annotated as a serine protease in the respective GenBank file, seems to be a homologue to heat shock protein HtrA from *Escherichia coli*. Its active site is fully conserved, and the extracellular localization has been determined previously [14]. The gene product of *hp1584* is annotated as a sialoglycoprotease (gcp). Its amino acid sequence does not contain known export motifs, and the amino acid composition is predicted to be cytoplasmic. However, the PA-SUB and PSORTb predictors categorized the protein as extracellular (Table 1) based on the extracellular localization of the homologous o-sialoglycoprotein endopeptidase of *Mannheimia haemolytica* (SwissProt identifier GCP_PASHA), which also lacks an N-terminal targeting signal [28]. In fact, very recently Hp0657, Hp1012, Hp1019, and Hp1350 have been identified in the extracellular *H. pylori* proteome [29] indicating the high specificity of our bioinformatical prediction of hypothetical extracellular *H. pylori* proteases (Table 1).

Since we demonstrated that several caseinolytic proteases are secreted by *H. pylori* independently of functional T4SS, it is likely that other secretion systems exist. This is underlined by our observation that nine out of 14 genes either contain a signal peptide, which only explains a transportation to the periplasm, or receive a high SecP prediction score (Table 1). We stress that these predicted features are common for extracellular proteins but do not explain a possible transport pathway. Thus one can speculate that a secretory machinery not yet attributed to *H. pylori*, or entirely novel ones, might be involved which require export signals of an unknown nature. For example, *H. pylori* might involve a specific type I (ABC) or a type III transportation system.

### *H. pylori* HtrA is an active protease

**Figure 2:**
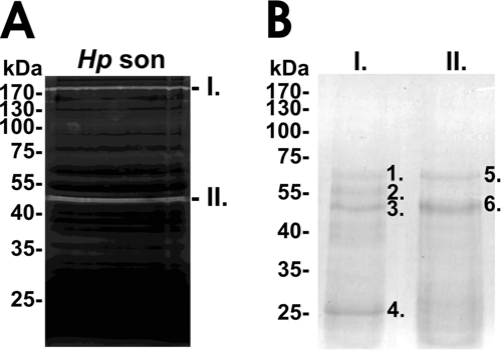
Identification of *H. pylori* proteases. (A) For a preparative analyses, 18×10^9^ bacteria were lysed and analyzed by zymography. The upper (1) and lower (2) negatively stained protease bands were excised, proteins were eluated and separated by SDS PAGE and Coomassie staining (B). Indicated protein bands were analyzed by mass-spectrometry.

We were then interested in answering the question whether one of the predicted *H. pylori* proteases accounts for the observed proteolytic activity. In a first step, concentrated *H. pylori* lysates were separated by zymography under non-reducing conditions followed by protein eluation of proteins from the negatively stained protein bands I and II (Figure 2A). Then, eluated proteins were concentrated and separated by a denaturating SDS PAGE (Figure 2B). We detected four different proteins in the Coomassie-stained SDS PAGE, which were isolated from protein band I in the zymogram (compare Figure 2A, band I and Figure 2B, lane I). Electrophoretic separation of proteins from protein band II (Figure 2A) resulted in two different proteins (Figure 2B, lane II). The identity of these proteins was determined by MALDI-TOF-MS. The accession number, denomination and a summary of the MS data are presented in Table 2. The results of the MS analyses are shown for a single database entry for each band. However, due to the high degree of sequence identity between proteins isolated from different *H. pylori* strains significant hits were obtained also for other urease and serine proteases, *e.g.* serine protease from *H. pylori* strain J99 or Ure B from database entry gi/51989332.

**Table 2:** Proteins that were identified by mass-spectrometry (*cf.* Figure 2B).

Band	Accession number	Protein name	Sample	Number of matched peptides	MASCOT Score	Sequence coverage
1	gi|19338960	urease B subunit [H.pylori]	A^a^	9	69^b^	25%
			B	8	98	20%
2	n.d.^c^					
3	gi|15645633	Serine protease (htra) [H.pylori 26696]	A	10	91	30%
			B	12	88	29%
4	n.d.					
5	n.d.					
6	gi|15645633	Serine protease (htra) [H.pylori 26696]	A	10	84	30%
			B	10	88	25%

^a^results of two independently processed samples; ^b^protein score was below the level that indicates a *p*-value of <0,05, ^c^not determined.

### Hp1018 encodes a signal peptide for an active Hp1019 protease

**Figure 3:**
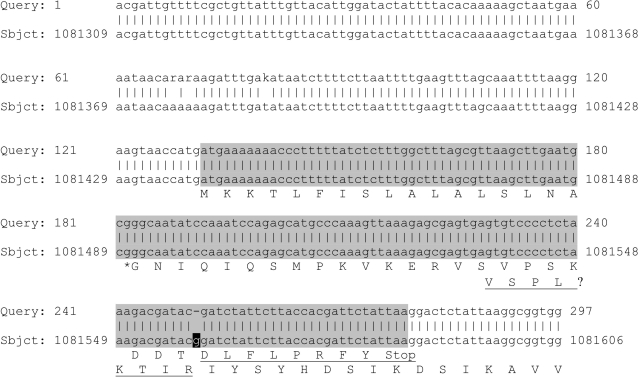
Blastn alignment of the re-sequenced nucleotide sequence (query) with the original genomic sequence (subject) of *hp1019*. The annotated gene *hp1018* is marked in grey. The letter ‘r’ represents (‘a’ OR ‘g’), while the letter ‘k’ represents (‘t’ OR ‘g’). The inserted guanidine is printed white on black. Numbers give residue positions. The amino acid translation is given in single letter code for Hp1018, starting at position 1081440, and for Hp1019, starting at position 1081537. The predicted most likely signal peptidase cleavage site between the amino acids LNA and GNI is marked with an asterisk. The underlined part of the amino acid sequences will not be part of the translation if the marked guanidine is removed.

Hp1019 has been previously predicted as a secreted *H. pylori* protease with unknown function [14], [29]. However, its proteolytic activity had not been demonstrated. Considering the protein sequence of *H. pylori* HtrA, it lacks an annotated N-terminal signal peptide, in contrast to HtrA of *E. coli*. The gene *hp1019* has an N-terminal overlap with the adjoining gene *hp1018*, which is 147 bases long and in a different reading frame. It has been suggested before that those genes might belong together [30]. Thus, we re-sequenced the gene *hp1018* and aligned it to the published genomic data of *H. pylori* Hp26695 (Figure 3). Here, we demonstrate that *hp1018* reveals a wrongly sequenced guanidine at position 1081558 of the published genome of *H. pylori* strain 26695. We conclude from our data that the translation of Hp1018 actually contains a signal peptide-like sequence (SignalP *score*>0.99) at its N-terminus, and it is most likely that Hp1018 represents the N-terminal part of Hp1019 resulting in a new sequence with 475 amino acids.

**Figure 4:**
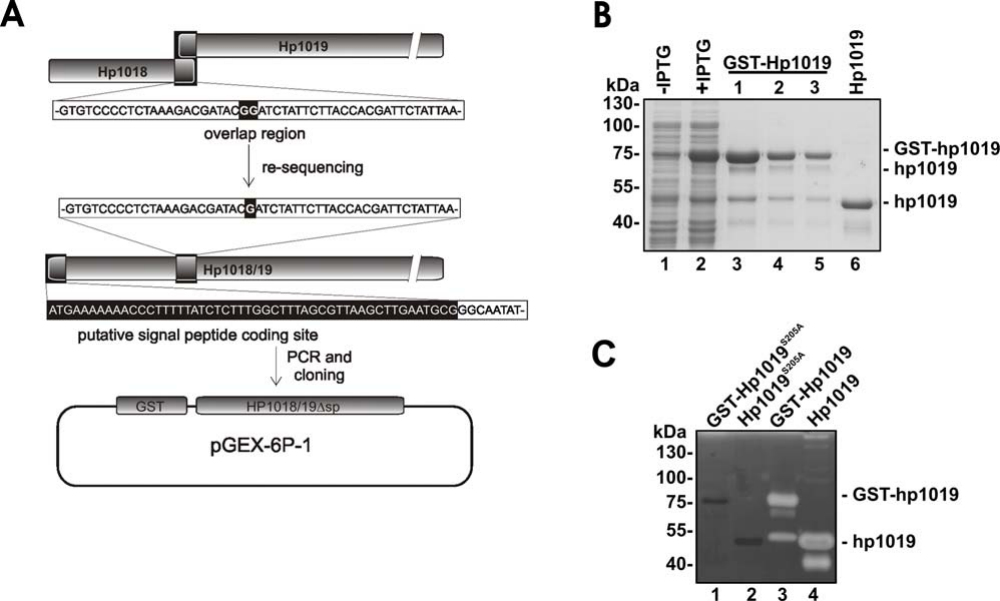
Proteolytic activity of the Hp1018/19 protein. (A) For the construction of the GST-Hp1018/19Δsp fusion protein, the re-sequenced Hp1018/19 gene was amplified without the putative signal peptide and cloned into the pGEX-6P-1 vector. (B) The *gst-hp1018/19Δsp* construct was transformed in *E. coli* for overexpression and total protein extracts from untreated (lane 1) and IPTG-induced *E. coli* (lane 2) were separated by SDS PAGE. Overexpressed GST-Hp1018/19Δsp was precipitated using glutathione sepharose and released by three eluation steps (lanes 3–5). To remove the GST tag, GST-Hp1018/19Δsp bound to glutathione sepharose were treated with PreScission protease and 30 µg protein of the supernatant containing the Hp1018/19 (lane 6) were loaded on a SDS PAGE followed by Coomassie staining. (C) Three µg of purified GST-Hp1018/19Δsp^S205A^ (lane 1), GST-Hp1018/19Δsp (lane 3), PreScission protease-treated Hp1018/19Δsp^S205A^ (lane 2) and Hp1018/19Δsp (lane 4) were analyzed by casein zymography for proteolytic activity.

To prove proteolytic activity of Hp1018/19 for the first time, we fused the *hp1018/19* gene lacking the putative signal peptide to the glutathione-S-transferase (*gst*) gene and transformed the construct into *E. coli* BL21 to express the recombinant protein (Figure 4A). Both, induction and enrichment of GST-Hp1018/19Δsp protein were analyzed by Coomassie-stained SDS PAGEs (Figure 4B). During GST-Hp1018/19Δsp preparation, contaminating proteins were co-purified, which were identified by MALDI-TOF-MS as glutathione-*S*-transferase and degradation products of HtrA. Accordingly, it had been demonstrated that *E. coli* encoded HtrA is an endopeptidase [31]. To remove the GST tag from the fusion protein, GST-Hp1018/19Δsp coupled to GST sepharose was incubated with PreScission protease resulting in the release of Hp1018/19Δsp protein (Figure 4B, lane 6).

Purified proteins were then probed for proteolytic activity (Figure 4C). The GST-Hp1018/19Δsp proteins were bound to GST sepharose, washed and eluated using reduced glutathione. As a control, we cloned and purified the Hp1018/19Δsp^S205A^ protein in which serine-205 was mutated to alanine in the presumable active center of HtrA. As expected, we observed casein degradation by GST-Hp1018/19Δsp protein (Figure 4C, lane 3), but not by the GST-Hp1018/19Δsp^S205A^ (Figure 4C, lanes 1–2). This finding demonstrates that *H. pylori* HtrA actually is an active protease, which can be inactivated by mutation of serine-205. In parallel, we cloned and purified Hp0506, Hp0657, Hp1012, Hp1037, Hp1543, and Hp0169, which previously had been described as a collagenase [13]. With the exception of Hp1019, we did not detect any proteolytical activities using casein as a substrate in zymography studies (data not shown). Therefore, we conclude that the observed caseinolytic activities were actually mediated by Hp1018/19.

As shown my mass-spectrometry, we also co-purified processed HtrA variants with GST-Hp1018/19Δsp (Figure 4B). We detected proteolytic activity of these proteins in casein zymography (Figure 4C). We therefore assume that processed variants of HtrA formed multimers with GST-Hp1018/19Δsp during the purification steps. This suggestion is supported by the finding that removal of the GST tag from GST-Hp1018/19Δsp protein led to the formation of the 170 kDa protease (Figure 4C, lane 4), which was not detected after purification of Hp1018/19Δsp^S205A^ (Figure 4C, lane 2). Together with our analysis showing that HtrA was present in the upper and lower protein bands (Figure 2), we conclude from our data that HtrA might also be active as a multimer.

### Conclusions

The complex mechanisms how *H. pylori* strongly induce inflammatory responses and invasive growth leading to the disruption of the human epithelium are still unclear. Although exported proteases of pathogens represent extensively studied virulence factors, not much is known about their involvement in *H. pylori*-associated pathogenesis. This comprehensive analysis of the *H. pylori* strain 26695 genome by sequence analysis and activity prediction methods revealed several genes coding for putative proteases. Among those, we identified the HtrA from *H. pylori* as a secreted enzyme exhibiting proteolytic activity. We also found that HtrA forms proteolytically active multimers, which is consistent with an earlier report of Windle and colleagues who demonstrated that *H. pylori* secretes a metalloprotease with a native molecular size of approximately 200 kDa and speculated whether this metalloprotease activity may be involved in proteolysis of a variety of host proteins *in vivo* and thereby contributes to gastric pathology [12]. The *E. coli* homologue HtrA functions as a heat shock protein, although it cannot be excluded that Hp1019 represents a so-called “moonlighting” protein [32], serving a function both in the periplasm in heat shock degradation and the extracellular matrix as a virulence factor. In fact, a secreted collagenase Hp0169 was identified as an important virulence factor for *H. pylori* colonization [13]. Although the biological function of Hp0169 and the recently detected extracellular proteases Hp0657, Hp1012, and Hp1350 [29] are unknown, it underscores the potential importance of secreted bacterial proteases in *H. pylori* mediated pathogenesis, which represent attractive vaccine and drug target candidates.

## Materials and Methods

### Homology search

Proteases were compiled for the reference data set by selecting all entries from the UniProtKB/SwissProt database (version 6.7) [18] containing the keyword “protease”, but lacking the phrases “inhibitor*”, “probable*”, “fragment*”, “hypothetical*”, “putative*”, “possible*” or “predicted*” in the keyword and description fields, where the asterisk is a wildcard for any arbitrary suffix. The NCBI BLAST package was employed for pairwise sequence alignment [19]. The Blastp program was used for protein-protein comparison. Tblastn was used to compare the whole DNA sequence of *H. pylori* strain 26695 with the protease sequence set. The substitution matrices PAM30, PAM70, PAM250, BLOSUM45, BLOSUM62 and BLOSUM80 were used with default parameter settings (*e.g.* scoring penalties, window size).

### Bacteria

*H. pylori* wildtype strains 26695 and P12, its isogenic mutant strains ΔVacA, and ΔPAI had been described before [11], [33]. Bacteria were grown in protein-free liquid brain heart infusion (BHI) medium (Merck, Darmstadt, Germany) supplemented with β-cyclodextrin for 48 hours, which has been previously optimized for minimal autolysis of *H. pylori* cells [14]. Lysates of *H. pylori* were obtained by sonification in PBS containing 0.1% Triton X-100. Supernatants of *H. pylori* BHI cultures were sterilized by filtration (pore size 0.22 µm).

### Amplification and sequencing of hp1018

The gene *hp1018* was amplified from the genomic DNA of *H. pylori* strain 26695 by standard PCR using the Pfx DNA-polymerase (Invitrogen, Karlsruhe, Germany). The following primers were used: hp1018for: 5′-GGC TAT GGA TAA GGA TCA ACG C-3′, hp1018rev: 5′-CCA CCG CCT TAA TAG AGT CCT T-3′. The PCR product, having a calculated length of 333 bases, was submitted to a commercial provider (GENterprise, Mainz, Germany) for sequencing.

### Cloning, mutation and purification of HtrA

The construct Hp1018/19Δsp was amplified from genomic DNA of *H. pylori* strain 26695 using the primers 5′-aaggatccggcaatatccaaatccagagcatg-3′ and 5′-aagaattcgacccacccctatcatttcacc-3′ with Pfx DNA polymerase in supplied buffer with 2× PCR Enhancer (Invitrogen). The amplified BamH1/EcoR1 flanked PCR product was then ligated into the pGEM-T Easy plasmid (Promega), subcloned into the pGEX-6P-1 plasmid DNA (GE Healthcare Life Sciences) and transformed in *E. coli* BL21. The construction of the protease-inactive Hp1018/19Δsp^S205A^ protein, serine 205 was mutated to alanine using the QuikChange® Lightning Site-Directed Mutagenesis Kit (Stratagene) according to the manufacturer's instructions. For heterologous overexpression and purification of GST-Hp1018/19Δsp, transformed *E. coli* was grown in 500 ml TB medium to an OD_550_ of 0.6 and the expression was induced by the addition of 0.1 mM isopropylthiogalactosid (IPTG). The bacterial culture was pelleted at 4000×g for 30 minutes and lysed in 25 ml PBS by sonification. The lysate was cleared by centrifugation and the supernatant was incubated with glutathione sepharose (GE Healthcare Life Sciences) at 4°C over night. The fusion protein was either eluted with 10 mM reduced glutathione for 10 minutes at room temperature or cleaved with 180 U Prescission Protease for 16 h at 4°C (GE Healthcare Life Sciences). Elution and cleavage products were analyzed by SDS PAGE and zymography.

### Zymography and protein eluation

Undiluted aliquots were loaded onto 8% SDS-PAGE containing 0.1% casein (Invitrogen, Germany) and separated by electrophoresis. After separation, the gel was re-naturated in 2.5% Triton X-100 solution at room temperature for 60 min with gentle agitation, equilibrated in developing buffer (50 mM Tris-HCl, pH 7.4; 200 mM NaCl, 5 mM CaCl_2_, 0.02% Brij35) at room temperature for 30 min with gentle agitation, and incubated overnight at 37°C in fresh developing buffer. Transparent bands of caseinolytic activity were visualized by staining with 0.5% Coomassie Blue R250. For identification of proteases present in zymographies, negatively stained bands were excised from a preparative casein zymogram and proteins were eluted twice for 6 hours *via* D-Tube™ Dialyzers Maxi MWCO 6–8 kDa (Novagene). Eluated proteins were desalted and concentrated using Vivaspin columns from Sartorius (Germany).

### Mass spectrometry

Eluated proteins from zymograms were separated by means of SDS-PAGE and stained with Coomassie for subsequent MS analysis in two independent experiments (A+B). In gel digestion was performed as previously described with several minor modifications [34]. Peptide mixtures were additionally purified and concentrated by using ZipTipC-18 tips (Millipore) according to the manufacturers' instructions. The identity of HtrA and urease B was proven by mass spectrometry as described [35], [36]. Briefly, Samples were mixed with peptide standard (peptide standard II, Bruker) and matrix (a saturated solution of HCCA in 50% ACN+0,5%TFA) at a ratio of 1∶1∶2; v∶v∶v), and with matrix only at a ratio of 1∶1; v∶v, and transferred on a ground steel target. Mass analysis was done on a Bruker Reflex II mass spectrometer with predefined default instrument settings. Proteins were identified by running MASCOT (http://www.matrixscience.com) against the entire NCBI database. Peptide tolerance was set to 50 ppm and a maximum of one missed cleavage site was allowed. A hit was considered as significant at a probability value of *p*<0.05.
